# On Medical Reform

**Published:** 1809-12

**Authors:** 


### To the Editors of the Medical and Phyfical Journal.

Gentlemen,

Having read in your Journal, for October last, a circular Letter from Dr. Harrison on the subject of Medical Reform, which appears to me, in some respects, to convey erroneous notions, and likely, if the proposed regulation in regard to physicians should be carried into effect, to render the qualification for practising in that department *looser*, instead of their being more strict, than before, I think it my duty to endeavour to obviate the impressions which the Doctor's letter is calculated to produce, and which his own well known candour would, I have no doubt, prompt him to counteract, if he were aware of his error.

With respect to the opinion of Mr. Serjeant Williams, on which the declaration concerning the Royal College of Physicians is said by Dr. Harrison (page 330 of your Journal) to be chiefly founded, it would be sufficient perhaps, in order to demonstrate its incorrectness, to oppose that of the present Lord Chief Justice of the Court of Common Pleas, Sir James Mansfield; which, being delivered from the bench, must (independently of the exalted character of that venerable person, as a lawyer) carry much higher authority than the learned Serjeant's. But I beg leave to go a step further, and to quote the very words of a clause of an existing act of parliament, which are too precise to need legal interpretation. The clause to which I allude is in an act of the 14th—15th of Henry the 8th, c. 5, and runs thus:

"And where [whereas] that in diocesys of England out of London, it is not light to fynde alway men hable to sufficiauntly examyne after the statute such as shall be admitted to exercyse Physyke in them, that it may be enacted in this present Parliament, That noo person from hensforth be suffered to exercyse or practyse in Physyke through England, untill such time that he bee examined at London by the said President and three of the said Electys: And to have from the said President or Electys, Letters Testimonials of theire approveing and examination, Except he be a Graduat of Oxford or Cantabrygge, which hath accomplished all thing for his fourme without any grace."

Now, Gentlemen, though I am no lawyer, I think it is pretty clear, that any person practising as a physician in any part of England out of London, and the distance of seven miles therefrom, (over which district there is the jurisdiction of the *Censors* of the College of Physicians, constituted by an earlier act of parliament) without being able to produce the testimonial letters of the *President* or *Elects* of the College, unless he be a graduate of Oxford or Cambridge, is guilty of a misdemeanour, and liable to be punished, in due course of law, by fine, imprisonment, or otherwise. It may be very true, that (to use the words of Mr. Serjeant Williams, as quoted by Doctor Harrison) "he is not under the controul, or supervision, or correction of the College of Physicians;" that is, the College cannot proceed against him, or exercise authority over him, in the same manner as they are empowered by law to do with respect to what may be called the *home* practitioners; but I am informed from as high a legal quarter as that which is referred to by Dr. Harrison, that the power of bringing such a person to justice is much more generally diffused, and that he is as much at the mercy of the meanest of the King's subjects, as a man who offends against any other statute, being equally liable to information or indictment. But, before I proceed any further, I will lay before you the opinion of Sir James Mansfield, to which I adverted above, and which I have extracted from an account of an Action in the Court of Common Pleas, taken in short hand by T. Jenkins, and published at Stafford.

"Sittings before Chief Justice Sir James Mansfield, and a Special Jury of Merchants, in Guildhall, London, on Tuesday the 22d of December, 1806.

"MIDDLETON v. HUGHES.

"*Chief Justice.* There was a great question, some time ago, agitated in the Court of Common Pleas about producing a diploma. You will find it said by the Judges in the Report of that Case, that a diploma confers no sort of authority whatever on the person holding it to act as a physician. It is one of the strangest things in the world, that questions should be raised upon a subject which has been so fully agitated already, and on which the decision is clear. This diploma, supposing the plaintiff to have one, gives no authority whatever to him to practise as a physician. The law of Harry the 8th is as clear as law can be, which is, that no man can practice as a physician, unless he has undergone the examination, and and met the approbation expressed by certificate, of certain members of the College of Physicians; and that if he does practice without it, he is liable to prosecution by indictment for certain penalties which he is thereby declared to have incurred, unless he be a graduate of Oxford or the other University. Now, the modern practice has been, to get a diploma from a Scotch University, and which is obtained in a very easy way, without the performance of any exercise, or the undergoing of any examination at all; a mere grant of favour, without any proof of fitness in the object of it: and it is a favour which the English Universities have no power to grant, if they were to attempt it. But it has been supposed that there is some virtue in these Diplomas from the Scotch Universities, because the Articles of Union have given them power to confer such a privilege, according to their discretion. But there is no such thing; a Diploma is an instrument which confers no authority whatever on the person possessing it."

If this clear and gravely delivered opinion have that weight with Dr. Harrison, which I think it *must* have, as soon as he is acquainted with it, through the medium of your Journal, (for I conceive that it has not hitherto come to his knowledge) I beg leave to submit to him, and to the profession at large, whether by the proposal, "that no unadmitted practitioner shall be suffered to act as physician, unless he be a graduate of some University" being carried into effect, a very salutary existing law would not be virtually abolished, and the public left at the mercy of any possessor of a purchased piece of parchment called a diploma, without the possibility of redress. I take the liberty also of submitting to Dr. Harrison's consideration another question, viz Whether, by attempting to reduce the graduates of all the Universities of the United Kingdom to the same level in the eye of the law, and to take away a tribunal for examination of *out* practitioners, which at present exists in the President and Elects of the London College, (and which I conceive to be a very wholesome one) he and the other advocates for the intended bill be not likely to call forth a very formidable opposition to that bill from certain powerful public bodies, who may, probably, feel themselves bound to endeavour to *maintain* and *retain* all their ancient privileges, and very strenuously too, such as they can demonstrate to be of public utility?

There is still one other question, which I hope it will

Mm 3 not not appear impertinent to offer, namely, whether the Lincolnshire Society would not be thought to confer a signal service on the community, by preferring indictments against such persons, as they can discover to be practising a most dignified and important branch of the medical profession, without having submitted themselves to the inquisition of fitness ordained by law?

I am, &c.

A LICENSED PHYSICIAN.

*Nov.* 20, 1809.

